# Elevated Pressure Increases Ca^2+^ Influx Through AMPA Receptors in Select Populations of Retinal Ganglion Cells

**DOI:** 10.3389/fncel.2018.00162

**Published:** 2018-06-13

**Authors:** Xiangyi Wen, Asia L. Cahill, Cody Barta, Wallace B. Thoreson, Scott Nawy

**Affiliations:** ^1^Department of Ophthalmology and Visual Sciences, Truhlsen Eye Institute, University of Nebraska Medical Center, Omaha, NE, United States; ^2^Department of Pharmacology and Experimental Neuroscience, University of Nebraska Medical Center, Omaha, NE, United States

**Keywords:** AMPA receptor, ganglion cell, retina, receptor plasticity, Ca^2+^ imaging, patch clamp, elevated pressure

## Abstract

The predominate type of AMPA receptor expressed in the CNS is impermeable to Ca^2+^ (CI-AMPAR). However, some AMPA receptors are permeable to Ca^2+^ (CP-AMPAR) and play important roles in development, plasticity and disease. In the retina, ganglion cells (RGCs) are targets of disease including glaucoma and diabetic retinopathy, but there are many types of RGCs and not all types are targeted equally. In the present study, we sought to determine if there are differences in expression of AMPARs amongst RGC subtypes, and if these differences might contribute to differential vulnerability in a model of stress. Using cultured RGCs we first show that acute exposure to elevated pressure increased expression of Ca^2+^-permeable AMPA receptors (CP-AMPARs) in some, but not all classes of RGCs. When RGCs were sampled without regard to subtype, AMPA currents, measured using patch clamp recording, were blocked by the CP-AMPAR blocker PhTX-74 to a greater extent in pressure-treated RGCs vs. control. Furthermore, imaging experiments revealed an increase in Ca^2+^ influx during AMPA application in pressure-treated RGCs. However, examination of specific RGC subtypes using reporter lines revealed striking differences in both baseline AMPAR composition and modulation of this baseline composition by stress. Notably, ON alpha RGCs identified using the Opn4 mouse line and immunohistochemistry, had low expression of CP-AMPARs. Conversely, an ON-OFF direction selective RGC and putative OFF alpha RGC each expressed high levels of CP-AMPARs. These differences between RGC subtypes were also observed in RGCs from whole retina. Elevated pressure further lowered expression of CP-AMPARs in ON alpha RGCs, but raised expression in ON-OFF and OFF RGCs. Changes in CP-AMPAR expression following challenge with elevated pressure were correlated with RGC survival: ON alpha RGCs were unaffected by application of pressure, while the number of putative OFF alpha RGCs declined by approximately 50% following challenge with pressure. Differences in expression of CP-AMPARs between RGC subtypes may form the underpinnings for subtype-specific synaptic plasticity. Furthermore, the differential responses of these RGC subtypes to elevated pressure may contribute to the reported resistance of ON alpha, and susceptibility of OFF and ON-OFF RGCs to injury in models of glaucoma.

## Introduction

AMPA receptors are tetramers, composed of combinations of four subunits, termed GluA1-A4. The GluA2 subunit is of critical importance as incorporation of this subunit is thought to eliminate Ca^2+^ permeation through the channel (Hollmann et al., 1991; Burnashev et al., 1992b), provided that the subunit has undergone editing at the Q/R site (Sommer et al., 1991). Thus Ca^2+^ permeability through a population of AMPARs can be augmented by reducing the number of edited GluA2 subunits, by removing the GluA2 subunit from the receptor completely, or by a combination of both. The vast majority of AMPARs expressed in the brain express the edited form of GluA2 and are therefore impermeable to Ca^2+^ (Isaac et al., 2007; Lu et al., 2009; Hanley, 2014). In brain, expression of CP-AMPARs is thought to be transiently expressed in principal neurons, associated with the induction phase of AMPAR LTP (Plant et al., 2006; Guire et al., 2008), or chronically expressed in interneurons such as cerebellar stellate/basket cells, where they are subjected to activity-dependent regulation (Liu and Cull-Candy, 2000; Bats et al., 2013). In the retina, expression of CP-AMPARs may be more common (Diamond, 2011). They are known to be expressed by OFF bipolar cells (Gilbertson et al., 1991), multiple types of amacrine cells (Morkve et al., 2002; Singer and Diamond, 2003; Chávez et al., 2006) and ganglion cells (Jones et al., 2012, 2014).

A common feature of many neurodegenerative diseases is an epigenetic reprogramming of AMPARs, resulting in an increase in Ca^2+^ permeability (Pellegrini-Giampietro et al., 1997; Wright and Vissel, 2012; Hwang et al., 2013; Yamashita and Kwak, 2014). Recent evidence suggests that an increase in Ca^2+^-permeable AMPARs may be a part of the etiology leading to loss of retina ganglion cells (RGCs) in models of elevated pressure and ischemia (Wang et al., 2014; Cueva Vargas et al., 2015; Park et al., 2016). Given the role of AMPARs in pathology of neurons that has been demonstrated elsewhere, we set out to determine if differences in AMPAR expression between RGC subtypes could be demonstrated. Furthermore, we wanted to test the idea that AMPAR expression could be further modulated by acute elevated pressure. A number of studies, carried out largely in humans or non-human primates before selective genetic markers for RGCs were available, suggested that cells with larger somas or axons are more susceptible to elevated pressure (Quigley et al., 1987, 1988; Glovinsky et al., 1991; Quigley, 1999; Shou et al., 2003). More recently, several studies taking advantage of genetic labeling of specific RGC populations have consistently demonstrated that OFF type RGCs show greater susceptibility to elevated IOP than their ON counterparts, including but not limited to loss of dendritic density, length and postsynaptic densities, and a reduction in light-evoked responses (Della Santina et al., 2013; El-Danaf and Huberman, 2015; Ou et al., 2016; Della Santina and Ou, 2017). Although the difference in susceptibility to IOP between the two pathways is clear, the underlying factors that contribute to this selectivity are completely unknown. It seems likely that molecular, rather than gross morphological differences between RGC subtypes are responsible for damage at early stages of degeneration following elevated IOP.

Here, we first show that when sampled randomly, RGCs increase expression of CP-AMPARs following exposure to elevated pressure. However, when individual RGC subtypes were probed using genetically labeled reporter lines, we found tremendous variability between subtypes in baseline levels of CP-AMPAR expression and response to elevated pressure. In particular, ON alpha RGCs express low levels of CP-AMPARs, and these levels remain low following acute elevation of pressure. Conversely, a subtype of ON-OFF direction selective RGC and an OFF RGC both expressed high levels of CP-AMPARs. Our results implicate AMPARs as a potential mediator of RGC death in glaucoma, and may explain the selective survival of RGCs in the face of elevated IOP. More generally, understanding the conditions that lead to changes in AMPAR expression will provide insight into general mechanisms of neurodegeneration, applicable not only to glaucoma, but to other blinding diseases where changes in AMPAR composition have been implicated, such as diabetic retinopathy (Barber et al., 2005; Kern and Barber, 2008; Castilho et al., 2015a,b).

## Materials and Methods

### Preparation of Cultures and Isolated Retinas

Animals were given food and water *ad libitum* and kept on a 12 h light-dark cycle. All procedures were approved by the University of Nebraska Medical Center Institutional Animal Care and Use Committee. To prepare cultures, retinas were isolated from newborn (P0) mice (C57BL/6J; RRID:IMSR_JAX:000664) after cryoanesthesia. Retinas were incubated for 45 min at 37°C in DMEM with HEPES, supplemented with 6 units/ml papain and 0.2 mg/ml cysteine. Papain was then inactivated by replacing the enzyme solution with complete media. Retinas were triturated through a fire-polished Pasteur pipette, plated onto glass coverslips pretreated with poly-D-lysine (0.1 mg/ml), and maintained in complete media at 37°C and 5% CO_2_ in a humidified atmosphere. Subsequently, every 3rd day, 50% of the culture medium was exchanged for fresh media. Complete media was composed of DMEM, 0.1% Mito^+^ serum extender, 5% heat-inactivated fetal calf serum, 1.0% penicillin-streptomycin-glutamine mix, BDNF (50 ng/ml), CNTF (20 ng/ml) and forskolin (5 μM). To prepare cultures of reporter lines, cultures from retinas of C57BL/6J were prepared as described above and allowed to grow to confluence in 7–10 days. Retinas from transgenic newborn mice were then dissociated and plated onto confluent C57BL/6J-derived cultures. Thy1-YFP16 (RRID:MGI:5752579), Kcng4^cre^ (RRID:IMSR_JAX:029414), and the reporter lines Ai6 (RRID:IMSR_JAX:007906) or Ai14 (RRID:IMSR_JAX:007908) were purchased from Jackson Labs. The Trhr-EGFP line (RRID:MMRRC_03036-UCD) was a generous gift of Dr. Marla Feller, University of California, Berkeley, Berkeley, CA, USA. The Opn4^cre/+^ line (Ecker et al., 2010) was a generous gift of Dr. Matthew Van Hook, University of Nebraska Medical Center. Cells were used for recording or immunohistochemistry at 4–28 days *in vitro*.

For patch clamp recording, coverslips were transferred to the recording chamber and bathed in a solution containing 137 mM NaCl, 28 mM glucose, 10 mM HEPES, 2.5 mM KCl, 2.5 mM CaCl_2_, 1 mM MgCl_2_, pH 7.4 with NaOH. Picrotoxin (100 μM) was added immediately before use. This same solution was used for dissection of isolated retinas described below. AMPA (100 μM) was delivered to cells by pressure ejection (Picospritzer) at 4–8 psi for 200 ms through a pipet with a resistance of 2–3 MΩ. PhTX was dissolved in the bath solution immediately before use and delivered through a local perfusion system (Warner Instruments) consisting of computer-controlled valves, a manifold into which the drugs flowed, and a fused silica capillary tube flowpipe (ID: 200 μm, Polymicro Technologies) at the output of the manifold which was positioned next to the recorded cell.

To pressure treat RGCs, coverslips housed in 12 or 24 well plates were placed in a custom-made chamber inside a 37°C incubator and pressure was increased to 40–47 mm Hg. A soldering iron was used to make holes in the tops of the plates. The chamber was fashioned by modifying a radioisotope storage chamber (Mitchell Plastics, RP-300) to accommodate inflow and outflow of a humidified gas mixture (95% air, 5% CO_2_), and a pressure gauge. A critical step in the modification was the addition of a rubber gasket and a series of screws around the perimeter to insure a tight seal of the chamber. Gas was delivered by a series of step down regulators to insure that pressure could be accurately controlled.

Experiments using isolated retinas were performed on mice of either sex aged 14–28 days. To prepare isolated retinas, mice were killed by inhalation of CO_2_ followed by cervical dislocation. Retinas were dissected free in a HEPES-based solution and incubated in HEPES solution supplemented with 240 units/ml collagenase and 2 mg/ml hyaluronidase. Retinas were then rinsed 3×, transferred to the recording chamber and kept in place with a slice anchor (Warner Instruments). All manipulations were performed in room light. Retinas were bathed in Ames media bubbled with 95% O_2_/5% CO_2_ at a flow rate of 3–5 ml/min at room temperature. AMPA and PhTX were delivered to RGCs in the intact retina using a puffer pipet and flowpipe apparatus as described for cultured cells. The flowpipe contained the HEPES-based solution to which PhTX (100 μM or 5 μM) and Cd^2+^ (100 μM, to block synaptic transmission) were added.

### Patch Clamp Recording

The recording chamber was mounted to an Olympus BX51 WI microscope and viewed with a 40× objective. Fluorescence for cell identification was provided by a 100 W Xenon source and EYFP (49029) and TdTomato (49004) filter sets (Chroma). For patch clamp recording of RGCs in culture or isolated retina, pipets were pulled to resistances of 4–8 MΩ and filled with a solution containing 123 mM gluconic acid (50% solution), 10 mM EGTA, 10 mM HEPES, 10 mM Glucose, 8.0 mM NaCl, 1.0 mM CaCl_2_, 4.0 mM Mg^2+^ATP and pH 7.4 with CsOH (50% solution). RGCs were held at −60 mV, including a measured junction potential of −10 mV. Series resistance was less than 20 MΩ and was not compensated for. Recordings were made with a Multiclamp 700B or 200B amplifier (Molecular Devices) and digitized at 1–10 kHz (ITC-18, HEKA). Online data acquisition and offline analysis were carried out using Axograph X. PhTX-74, a synthetic analog of PhTX, a toxin found in wasp venom, was used to block CP-AMPARs. This analog blocks homomeric GluA2-lacking AMPARs, as do other polyamine toxins, but it also blocks GluA2-containing heteromeric AMPARs in a dose dependent fashion (Nilsen and England, 2007; Poulsen et al., 2014). During PhTX application, AMPA was applied for a series of trials, typically 20. PhTX showed use dependent cumulative block of AMPARs as reported elsewhere. Raw data showing block of PhTX are the average of trials 17–20. To unblock AMPARs, cells were stepped for 1 s to +70 mV during AMPA application for 20 trials. This approach allowed for measurement of a block by 100 μM and 5 μM PhTX in the same cell. These protocols were carried out for RGCs in both culture and isolated retina. AMPA and PhTX-74 were purchased from Tocris-R&D Systems. All other reagents were purchased from Sigma-Aldrich.

### Calcium Imaging

For Ca^2+^ imaging experiments, we used the high affinity Ca^2+^ indicator Rhod-2 to minimize overlap with GFP fluorescence in RGCs. RGCs were incubated for 30 min in Rhod-2-AM (5 μM) in incubator (37°C, 5% CO_2_). Cells were washed three times with external saline and allowed to rest 20 min before imaging. Cells were viewed through a 100×, 1.65 NA, oil-immersion objective on an inverted microscope (Olympus IX71). Excitation light was filtered through a 561 nm (14 nm wide) bandpass filter from a 120 W mercury lamp (XCite 120Q, Olympus, Japan). The emitted fluorescence of Rhod-2-AM was collected through a 609 nm (54 nm wide) bandpass filter. When imaging EGFP, fluorescence was excited through a 482 nm (18 nm wide) bandpass filter and collected through a 525 nm (45 nm wide) bandpass filter. Images were acquired through an EMCCD camera (Hamamatsu ImageEM) every 1 s (40-ms exposure) and analyzed using Metamorph software (Molecular Devices). Ca^2+^ transients were evoked by application of a 500 ms puff of AMPA (100 μM) applied through a glass patch pipette 10–20 μm away from the cell using a pressure valve system (Toohey Company; 8 psi). During measurements, cells were superfused with external saline and 100 μM Cd^2+^ was present in all bath solutions except otherwise noted to block voltage gated Ca^2+^ channels that would otherwise contribute to the transients as a result of membrane depolarization. PhTX was present in the bath if needed. Background fluorescence was determined from the average fluorescence measured in five different fields from a coverslip of cells that were not loaded with Rhod-2-AM. This average value for background fluorescence was subtracted from the fluorescence measured in Rhod-2-loaded cells. Baseline fluorescence (F) was averaged from the first six data points in each trial. The stimulus-evoked change in fluorescence (ΔF) was then used to calculate the ratio ΔF/F.

### Immunohistochemistry

Coverslips were fixed for 30 min in 5% paraformaldehyde, washed and permeabilized with 0.1% TritonX-100 in PBS for 5 min. Coverslips were then incubated in 5% goat serum and Tuj-1 (R&D Systems, 1:200) or SMI-32 antibody (EMD Milipore, 1:500) in PBS overnight at 4°C. Coverslips were wash 3× and treated for 1 h with goat anti-mouse IgG conjugated to FITC or Rhodamine-Red (Invitrogen, 1:100) and 5% goat serum in PBS. Coverslips were mounted in Prolong antifade media (ThermoFisher Scientific) and viewed on a Nikon Eclipse inverted microscope for photography and cell counting.

### Experimental Design and Statistical Analysis

Data are expressed as mean ± SEM and are indicated in the figure legends and results section. Statistical significance was determined using the Wilcoxon-Mann-Whitney test as this test does not assume a normal distribution of data. For patch clamp studies, *n* indicates the number of cells that were included in the statistical analysis. At least three separate cultures contributed to the total number of cells reported. Both sexes of newborn mice were used for generation of primary cultures. For cell counting and Ca^2+^ imaging experiments, *n* indicates the number of separate cultures or coverslips that were included in the statistical analysis. The *n* values are indicated in the figure legends.

## Results

### Elevated Pressure Increases Overall Expression of CP-AMPARs

**Figure 1:**
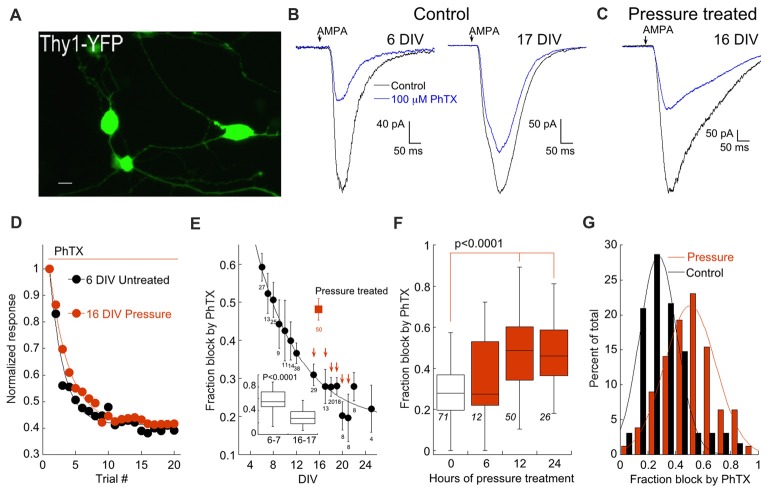
Elevated pressure increases expression of Ca^2+^-permeable AMPA receptors (CP-AMPARs). **(A)** Image of cultured retina ganglion cells (RGCs) from the Thy-1 YFP16 transgenic line. Scale bar: 10 μm. **(B)** Responses of RGCs to AMPA before during and after application of 100 μM PhTX. Cells are from a 6 days (left) and 17 days *in vitro* DIV, (right) cultures. **(C)** As in **(B)** except that the RGC is from a 16 DIV culture that was pressure-treated for 12 h. **(D)** Plot showing the fraction of block by PhTX with successive trials of AMPA application. The cells from **(B,C)** are depicted. Typically the effect of PhTX reached a plateau after about 10 trials. Continuous curves are single exponential fits to the data. Measurements of PhTX block in this and subsequent figures were made by averaging trials 17–20. **(E)** Summary of the block by 100 μM PhTX as a function of DIV. Solid square indicates pressure-treated RGCs (12 h). The number of sampled RGCs is indicated below each data point. Arrows indicate *p* = 0.00039, 0.00163, 0.00013, 0.00016, 0.00042, 0.00302 from DIV 15–21 (Wilcoxon-Mann-Whitney test) compared to pressure-treated cells. Inset: Box and whisker plot for RGCs at 6–7 (*n* = 44) and 16–17 DIV (*n* = 36). **(F)** Effect of pressure as a function of time. Number of cells is indicated. Control is from cells aged 15–18 DIV. Recordings were performed within 24 h after pressure treatment. **(G)** Histogram denoting the distribution of pressure-treated (12 h) and control (15–18 DIV) cells according to fraction of block by 100 μM PhTX. Fits are Gaussian distributions with peaks at 0.27 and 0.51, and standard deviations of 0.19 and 0.26 for control and pressure-treated, respectively.

Retinal ganglion cells (RGCs) were identified by use of the Thy1-YFP-16 line developed in the Sanes lab (Feng et al., 2000; Figure 1A). AMPAR currents were evoked by brief focal application of 100 μM AMPA. Responses are generated by activation of all (i.e., synaptic and nonsynaptic) AMPARs. To determine the contribution of CP-AMPARs to the total AMPAR current, we applied philanthotoxin-74 (PhTX) a synthetic analog of the wasp venom philanthotoxin (Kromann et al., 2002; Poulsen et al., 2014), a member of the arthropod family of toxins that block CP-AMPARs (Blaschke et al., 1993; Herlitze et al., 1993; Washburn and Dingledine, 1996; Toth and McBain, 1998). Two concentrations of PhTX were used. The lower concentration (5 μM) has been reported to block CP-AMPARs that lack GluA2 while the higher concentration (100 μM) also blocks GluA2-containing heteromeric receptors (Poulsen et al., 2014). To be certain that block of CP-AMPARs by PhTX reached steady state, AMPA was applied for 20 successive trials in the presence of the blocker. This number of trials were sufficient to produce a clear plateau in the amount of block (Figure 1D).

The fraction of AMPAR current blocked by 100 μM PhTX in RGCs was tightly linked to the age of the culture. For example, 100 μM PhTX blocked an average of 57 ± 3% of the total AMPA current at 6–7 DIV, but only 28 ± 2% of the current at 17–18 DIV (*p* < 0.0001; Wilcoxon-Mann-Whitney test; Figures 1B,E). An alternative hypothesis is that AMPAR expression is constant throughout development, but RGCs with low CP have a higher probability of survival. This seems unlikely, as the distribution of CP expression of RGCs early in development is narrow (Figure 1E, inset), necessitating a massive loss of RGCs to account for the shift in mean CP observed later in development. The developmental shift in the fraction of block by PhTX implies a change in surface expression of AMPARs by RGCs. Such a shift in the composition of AMPARs during development has been observed in other brain regions (Monyer et al., 1991; Pickard et al., 2000; Kumar et al., 2002).

To make comparisons between control and pressure-treated cultures more consistent, we performed pressure treatment experiments at roughly the same time points in culture (13–18 DIV). We constructed a pressure chamber following a previous design (Liu et al., 2007). Others have shown using both empirical methods (Sappington and Calkins, 2006; Liu et al., 2007) and theoretical considerations (Yang et al., 1993) that the increase in pressure has a negligible effect on pH or the concentration of gases dissolved in the media. Pressure was elevated to 40–47 mm Hg for durations between 6 h and 24 h. At a duration of 12–14 h, elevating pressure increased the fraction of current block by 100 μM PhTX to 48% compared to 29% in control cells, a highly significant difference (*p* < 0.0001; Figures 1C,E). Increasing the duration of pressure did not further increase block by 100 μM PhTX (47%, Figure 1F). Reducing the duration of pressure treatment to 6 h resulted in a 36% block of AMPAR by 100 μM PhTX, not significantly different from control. Thus, varying the duration produced an all or none threshold effect. Histograms of the pressure-treated (12 h) and control RGC distributions could both be well fitted with Gaussian functions of similar width and the primary effect of pressure was to shift the mean of the function (Figure 1G). Taken together, these results suggest that elevated pressure enhances expression of CP-AMPARs by RGCs.

**Figure 2:**
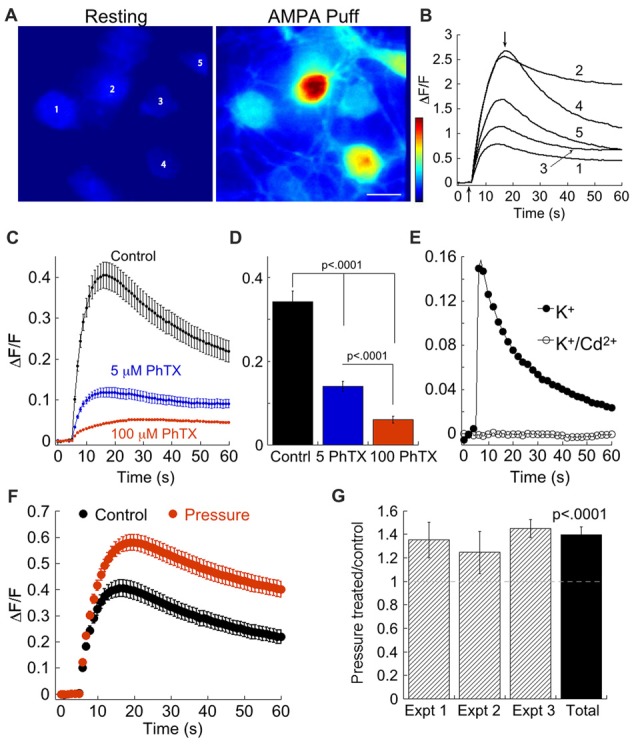
Imaging reveals an increase in Ca^2+^ influx following pressure treatment. **(A)** Heat map images of a cluster of RGCs before and during application of AMPA. **(B)** Time course of the fluorescence change (ΔF/F) in response to a 500 ms puff of 100 μM AMPA delivered 5 s after the start of the trace in the five RGCs depicted in **(A)**, labeled 1–5. Vertical arrows indicate the time points at which the two images in **(A)** were obtained. **(C)** Time course of the Ca^2+^ response to AMPA in the presence of 5 μM (*n* = 103) or 100 μM PhTX (*n* = 72), or in its absence (*n* = 114). As washout of PhTX is not complete without manipulation of the RGC membrane potential, application of 5 and 100 μM PhTX was carried out on separate cultures. Cells were not pressure-treated. **(D)** Summary of peak ΔF/F response to AMPA in control (209 cells, three experiments), 5 μM PhTX (103 cells, one experiment), and 100 μM PhTX (72 cells, one experiment). **(E)** Time course of the mean Ca^2+^ response to elevated K^+^ before (*n* = 4 cells) and during application of 100 μM Cd^2+^ (*n* = 4 cells). **(F)** Comparison of the Ca^2+^ transients in pressure-treated (*n* = 125) and control cells (*n* = 114) from the same culture. **(G)** Summary data from three separate experiments plotting mean peak ΔF/F normalized to control. For each experiment, ΔF/F of individual pressure-treated RGCs was normalized to the mean ΔF/F of control cells for that experiment: ΔF/F_pressure_/mean(ΔF/F_control_). The pressure-treated/control ratio was highly significant compared to unity (*n* = 181 pressure-treated and 188 control RGCs from three experiments; Wilcoxon-Mann-Whitney test, single group vs. 1). Experiment depicted in **(F)** is Experiment 3.

We tested the assertion that pressure elevates CP-AMPAR using a second independent approach, comparing intracellular Ca^2+^ transients in control and pressure-treated RGCs loaded with the Ca^2+^ indicator, Rhod-2. We first set out to isolate transients resulting from entry of Ca^2+^ through CP-AMPARs. Puffs of AMPA evoked increases in Ca^2+^ in clusters of RGCs downstream from the puffer pipet (Figures 2A,B). To block voltage-gated Ca^2+^ channels that open when RGCs are depolarized by AMPA, we added 100 μM Cd^2+^ to the bath. To demonstrate that this concentration of Cd^2+^ is effective, we first depolarized RGCs with K^+^ puffs to activate Ca^2+^ channels. Inclusion of 100 μM Cd^2+^ eliminated the K^+^-evoked Ca^2+^ transients, indicating this concentration was sufficient to block Ca^2+^ channels (Figure 2E). In the presence of Cd^2+^, but absence of PhTX (Figures 2C,D, control), AMPA evoked robust Ca^2+^ transients. These Ca^2+^ transients were largely blocked by 5 μM PhTX, indicating that the primary source of Ca^2+^ influx was homomeric CP-AMPARs (Figures 2C,D). Although significantly reduced by 5 μM PhTX, Ca^2+^ responses were still present, consistent with the presence of a population of AMPARs that are Ca^2+^ permeable but insensitive to low concentrations of PhTX. Exposure to 100 μM PhTX further decreased Ca^2+^ influx, and the difference was highly significant (*p* < 0.0001) compared with 5 μM PhTX. This result implies that heteromeric AMPARs constitute a measurable, albeit small source of Ca^2+^ influx in RGCs. It is presently unclear if these receptors have undergone Q/R editing of the GluA2 subunit. To determine if acute elevation of pressure increased Ca^2+^ influx through AMPARs, we measured Ca^2+^ transients in the presence of Cd^2+^. In pressure treated and sister untreated cultures at 15–16 DIV from three separate experiments, we found that elevated pressure (12–18 h) significantly (*p* < 0.0001) increased Ca^2+^ transients compared with untreated sister cultures (Figures 2F,G). Overall, pressure treatment increased Ca^2+^ influx by 35% compared to control (Figure 2G). Thus, changes in CP-AMPAR expression following transient pressure elevation can be detected by application of PhTX using both whole cell recording and by Ca^2+^ imaging.

To determine if pressure elevation impacted overall RGC survival, we counted RGCs labeled with the marker TUJ-1 5 days after beginning the pressure regime. Following pressure treatment the number of RGCs was reduced by 32.4 ± 8.7% compared with control sister cultures (*n* = 4 cultures, *p* = 0.008). Thus, elevated pressure up-regulated CP-AMPAR expression, increased Ca^2+^ influx through AMPARs, and significantly reduced survival of RGCs.

### RGC Subtypes Express Different AMPAR Compositions

**Figure 3:**
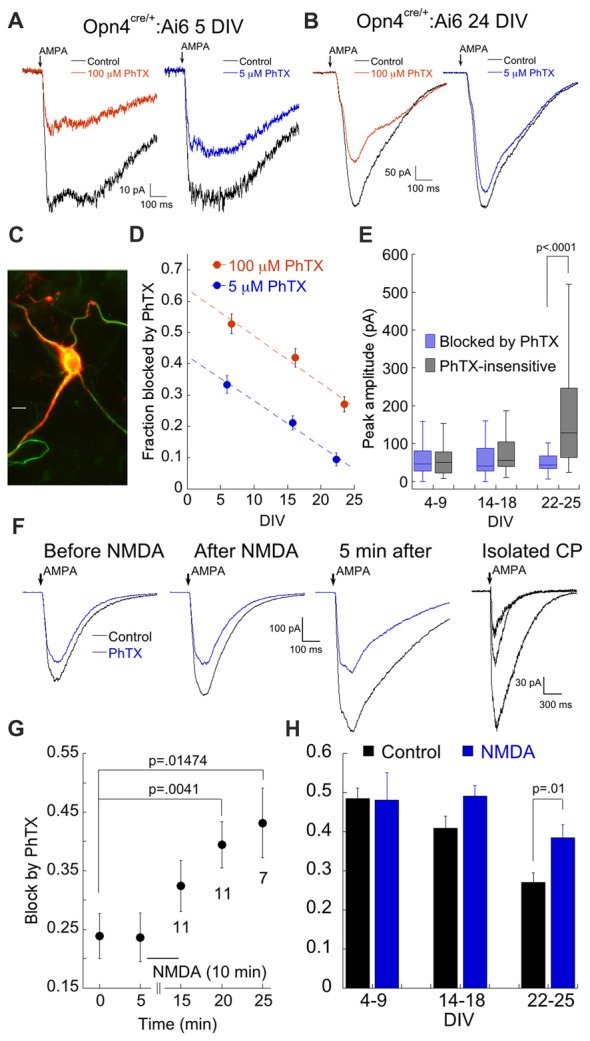
ON alpha RGCs express low levels of CP-AMPARs. **(A)** Examples of the response to AMPA in the presence of 100 μM and 5 μM PhTX in a fluorescent cell (5 DIV). **(B)** As in **(A)**, but from a 24 DIV culture. **(C)** Image of a EGFP-expressing cell labeled with SMI-32. Labeling by SMI-32 was a common characteristic of EGFP-expressing cells. Calibration bar: 10 μm. **(D)** Age dependent decrease in the fraction of presumptive GluA2-containing and GluA2-lacking CP-AMPARs probed with 100 μM and 5 μM PhTX, respectively. **(E)** Plot of the raw amplitudes of the AMPAR current blocked and insensitive to 100 μM PhTX, as a function of age in ON alpha RGCs. 4–9 DIV: *n* = 59; 14–18 DIV: *n* = 37; 21–25 DIV: *n* = 28. **(F)** Response of an ON alpha RGC to AMPA in control solution and 100 μM PhTX before and after application of 250 μM NMDA as indicated. Far right panel shows the isolated CP-AMPA current, obtained by subtraction, for all three time points. **(G)** Summary of the amount of block by 100 μM PhTX after NMDA application. Number of cells for each time point is indicated below the data points. **(H)** Summary of the contribution of the NMDA-mobilized pool of CP-AMPAR relative to total AMPAR as a function of age. For this experiment, RGCs were first exposed to NMDA or control solution for 10 min prior to break in and measurement of AMPA current. 4–9 DIV: *n* = 59 and 9 for control and NMDA, respectively. 14–18 DIV: *n* = 37 and 14. 22–25 DIV: *n* = 28 and 15.

Thus far we have examined the effects of elevated pressure on AMPAR expression in the overall population of RGCs. Next, we focused on specific subtypes using a reporter line strategy to probe for differences in AMPAR expression. We crossed Opn4^cre/+^ mice with loxP reporter lines expressing td-Tomato (Ai14) or EGFP (Ai6) to label melanopsin-expressing ipRGCs (Ecker et al., 2010; Estevez et al., 2012; Schmidt et al., 2014). Although the Opn4 line labels at least five types of melanopsin expressing ipRGCs (Ecker et al., 2010), only the ON alpha M4 RGC is labeled by SMI-32, an antibody directed against unphosphorylated neurofilaments (Schmidt et al., 2014; Sexton et al., 2015). Approximately 75% of fluorescent RGCs in our cultures were positive for SMI-32 (134/178; *n* = 3 cultures; Figure 3C). Thus, our culture conditions appear to favor ON alpha RGCs over other ipRGCs. Morphologically, labeled RGCs from the Opn4 line were characterized by a small number of relatively unbranched processes that extended for great distances beyond the field of view. Functionally, Opn4 RGCs often exhibit spontaneous excitatory synaptic potentials, a distinguishing characteristic, as other RGCs exhibited little or no spontaneous activity.

To measure CP-AMPAR receptor expression in ON alpha RGCs, we followed the same approach as before, evoking AMPA currents in the presence of 100 μM or 5 μM PhTX. As was observed in the overall population of RGCs, the fraction of block by PhTX decreased with age in labeled RGCs from the Opn4:EGFP line, and this was true for both concentrations of PhTX (Figures 3A,B,D). The ability to focus on a single class of RGC allowed us to directly compare the absolute amplitude of the (PhTX-sensitive) CP- and (PhTX-resistant) CI-AMPAR components at different developmental time points. Surprisingly, the amplitude of the CP-AMPAR component, measured with 100 μM PhTX, remained stable throughout development, while the CI-AMPAR component increased over time (Figure 3E). Thus, the decrease in relative expression of CP-AMPARs during development can be completely attributed to a dramatic increase in the number of CI-AMPARs. A similar observation has been made for developing neurons in other brain regions, including hippocampus (Pickard et al., 2000). These findings indicate that ON alpha RGCs exert remarkably precise control of CP-AMPAR expression over time.

We have previously shown that ON, but not OFF type RGCs mobilize internal CP-AMPARs for insertion to the surface via both homeostatic and NMDAR dependent mechanisms (Xia et al., 2007; Jones et al., 2012; Casimiro et al., 2013), and we wondered whether ON alpha RGCs labeled in the Opn4 line might similarly maintain a constant number of surface CP-AMPAR by removal and sequestration of excess receptors into an intracellular pool. To test this, AMPA currents were first recorded in the presence and absence of 100 μM PhTX to estimate the size of the surface pool of CP-AMPARs. NMDA (250 μM) was then applied for 10 min and the size of the surface pool of CP-AMPARs was re-measured (Figure 3F). While the CI-AMPAR component remained constant, the CP-AMPAR component was potentiated, consistent with the idea that they were rapidly recruited from an intracellular pool (Figure 3G). To improve efficiency, we modified the protocol and applied NMDA for 10–15 min to the coverslip prior to recording. Recordings were carried out within 30 min following NMDA treatment. NMDA-dependent plasticity was age dependent. It potentiated CP-AMPARs of ON alpha RGCs from cultures that were maintained for at least 21 DIV. Conversely, when tested on cultures of 4–9 DIV, NMDA had no effect on the CP-AMPA current (Figure 3H). These findings suggest that ON α RGCs maintain an increasingly large pool of intracellular CP-AMPARs as they develop, a pool that can be rapidly delivered to the surface following NMDA stimulation.

We generated a second mouse line (here called Kcng4) by crossing Kcng4^cre/-^ mice with the EGFP (Ai6) or tdTomato reporter line (Ai14). The Kcng4^cre/-^ line reportedly labels both ON and OFF alpha RGCs (Duan et al., 2014; Krieger et al., 2017). However, the number of labeled cells in the Kcng4 line was much greater than for the Opn4 line, which as discussed above, labels primarily ON alpha cells (Kcng4, 255 ± 11.35 cells/coverslip; Opn4, 33 ± 2.9 cells/coverslip, *n* = 6 coverslips, three cultures, 14 DIV). Assuming that equal numbers of ON alpha cells survive in each mouse line, and were randomly selected for recording, ON alpha cells would contribute less than 15% of the total labeled RGC population in the Kcng4 line. In line with this estimate, we found that spontaneous synaptic activity, a hallmark of ON alpha cells in the Opn4 line, was largely absent in labeled RGCs in the Kcng4 line. In addition, as will be discussed below, NMDA failed to increase CP-AMPAR currents, consistent with previous findings for OFF RGCs in *ex vivo* retina (Jones et al., 2012).

**Figure 4:**
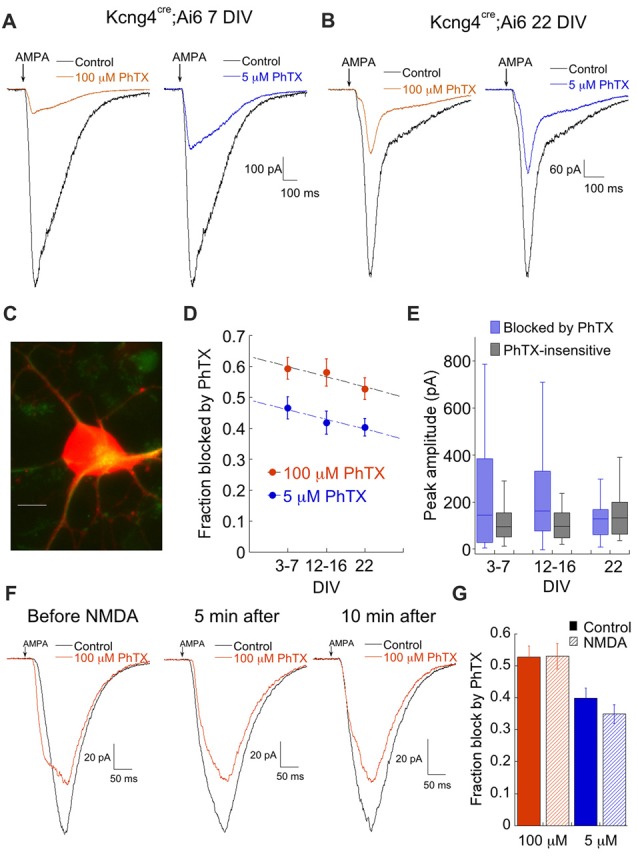
Putative OFF alpha RGCs express high levels of Ca^2+^ permeable AMPARs throughout development. **(A)** Representative responses to AMPA in the absence and presence of 100 μM or 5 μM PhTX from a putative OFF alpha RGC at 7 DIV, illustrating the large amount of PhTX block that is typical for these cells. **(B)** As in **(A)**, at 22 DIV. **(C)** Image of a fluorescent cell from a Kcng4^cre^; tdTomato mouse. The cell was also weakly labeled with SMI-32 (green). Scale: 10 μm. **(D)** Summary of fraction of AMPA current blocked by 100 μM and 5 μM PhTX as a function of culture age. 100 μM PhTX: *n* = 40, 27, 12 for 3–7 DIV, 12–16 DIV and 22 DIV, respectively. 5 μM PhTX: *n* = 26, 25, 11. **(E)** Summary of the amplitude of the PhTX-sensitive and insensitive components, determined using 100 μM PhTX, as a function of age. 3–7 DIV: *n* = 40. 12–16 DIV: *n* = 27. 22 DIV: *n* = 12. **(F)** Representative traces at 3 indicated time points demonstrating that NMDA (250 μM) had no effect on trafficking of CP-AMPARs to the cell surface. Similar results were obtained in 3 other cells. **(G)** Summary of the lack of effect of NMDA on responses to both 100 μM PhTX (control, *n* = 12; NMDA, *n* = 14) and 5 μM PhTX (control, *n* = 10; NMDA, *n* = 14). Experiments summarized here were carried out by pre-incubating cells in NMDA or control solution for 10 min prior to recording.

AMPAR currents in labeled cells from the Kcng4 line (Figure 4C) were strongly blocked by both 100 μM and 5 μM PhTX, particularly in younger cultures (Figures 4A,B). There was a slight tendency for the contribution of CP-AMPAR to decrease over time, but it was much less pronounced than the decrease observed in ON alpha RGCs (Figure 4D). Examination of the raw amplitudes of the CP- and CI-AMPAR components over time revealed a decrease in the CP-AMPAR component and an increase in the CI-AMPAR component, but neither change reached statistical significance (Figure 4E). We tested the effects of NMDA on CP-AMPAR expression. Contrary to ON RGCs, there was no effect of NMDA on CP-AMPAR expression in labeled cells from the Kcng4 line (Figures 4F,G). A lack of NMDA-dependent plasticity has been observed previously for OFF type RGCs in *ex vivo* retina (Jones et al., 2012). These data are consistent with the idea that ON RGCs maintain an intracellular pool of CP-AMPARs, while OFF type RGCs traffic most or all CP-AMPARs to the surface.

Thus, RGCs belonging to the Opn4 and Kcng4 genetic cohorts display very distinct expression of AMPARs: when measured using 100 μM PhTX, there was no significant difference in fractional expression of CP-AMPAR between the two groups at early culture stages (*p* = 0.32, DIV 3–8), but the difference was highly significant at later times (*p* = 0.0004, 12–18 DIV; *p* < 0.0001, 22–24 DIV; compare Figures 3, 4). When probed with 5 μM PhTX, the difference in expression of CP-AMPAR between these two groups was highly significant at all culture ages (*p* < 0.0001). This highly divergent expression of AMPA receptors may help ON RGCs limit excessive Ca^2+^ influx through CP-AMPARs and protect them from damage due to stressful events such as elevated IOP, while OFF RGCs may be more vulnerable.

**Figure 5:**
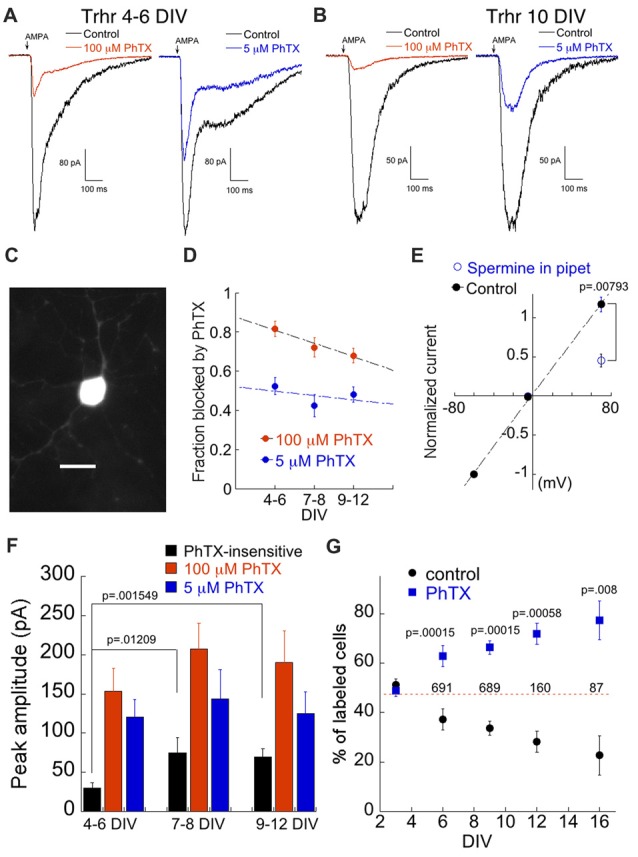
High expression of CP-AMPARs confers susceptibility to cell death in a population of DS-RGCs. **(A)** Examples of the AMPA response in labeled cells from the Trhr line in the presence of 100 μM (4 DIV, left) and 5 μM PhTX (6 DIV, right). Left and right traces are from two different cells. **(B)** As in **(A)** but from a 10 DIV RGC. **(C)** Image of a GFP positive cell at 7 DIV. **(D)** Summary of fraction of AMPA current blocked by 100 μM and 5 μM PhTX as a function of culture age. 100 μM PhTX: *n* = 16, 16, 19 for 4–6 DIV, 7–8 DIV and 9–12 DIV, respectively. 5 μM PhTX: *n* = 8, 8, 20. **(E)** I-V plot demonstrating spermine (500 μM)-dependent block of AMPA current at positive voltages. *n* = 5 for both control and spermine. **(F)** Amplitude of the PhTX-resistant AMPA component, and the components blocked by 5 μM and100 μM PhTX plotted as a function of age. Same cell population as in **(D)**. **(G)** Effect of 5 μM PhTX treatment on DS-RGC survival. Each culture was divided into control and PhTX-treated. For each experiment, two coverslips for each condition and time point were counted. Data are expressed as the percent of the total number of cells in each category, and are the mean and SEM of 5–7 separate experiments. The numbers above the dashed line correspond to the mean number of cells at that time point.

We examined a third mouse line, Trhr-EGFP (Trhr) in which a population of direction selective RGC (DS-RGC) that prefers motion in the posterior axis expresses EGFP (Rivlin-Etzion et al., 2011, 2012). EGFP-positive RGCs were smaller and had finer and shorter processes compared to labeled cells in the Opn4 and Kcng4 lines (Figure 5C). Sample recordings from two EGFP expressing cells are shown in Figures 5A,B. AMPAR currents were highly susceptible to block by PhTX, indicating high expression levels of CP-AMPAR. Application of PhTX revealed a large CP-AMPAR component that decreased only slightly over time (Figures 5A,B,D,F). The CP-AMPAR ratio of Trhr-EGFP positive cells, measured with either 100 μM or 5 μM PhTX, was comparable to the ratio for putative OFF alpha cells from the Kcng4 line (compare Figures 4D, 5D). As discussed above, low concentrations of PhTX-74 are thought to selectively block homomeric CP-AMPARs (Poulsen et al., 2014). As an independent verification of this, we added to the internal solution the polyamine spermine (500 μM), an internal blocker of homomeric, GluA2-lacking CP-AMPARs (Bowie and Mayer, 1995; Kamboj et al., 1995; Koh et al., 1995) and measured the rectification ratio at positive and negative holding potentials. We obtained a rectification ratio of 0.45 ± 0.083 (*n* = 5; Figure 5E), in good agreement with the amount of block measured using 5 μM PhTX, (48 ± 3.8%; Figure 5D). Thus the two approaches yield similar estimates of the fraction of current carried by homomeric CP-AMPARs.

One robust characteristic of the DS RGCs was a sharp decrease in survival, beginning after approximately 1 week in culture. After 2 weeks, nearly all of this RGC population was gone. To determine if expression of high levels of CP-AMPARs might contribute to the loss of DS RGCs, we chronically blocked these receptors with PhTX. Indeed, 5 μM PhTX significantly improved survival compared to control sister cultures beginning at 9 DIV (Figure 5G). By 16 DIV, 78% of the surviving cells were in the PhTX treated group. Thus, this population of DS RGCs is much more susceptible to CP-AMPAR-related cell death than ON and OFF alpha cells.

**Figure 6:**
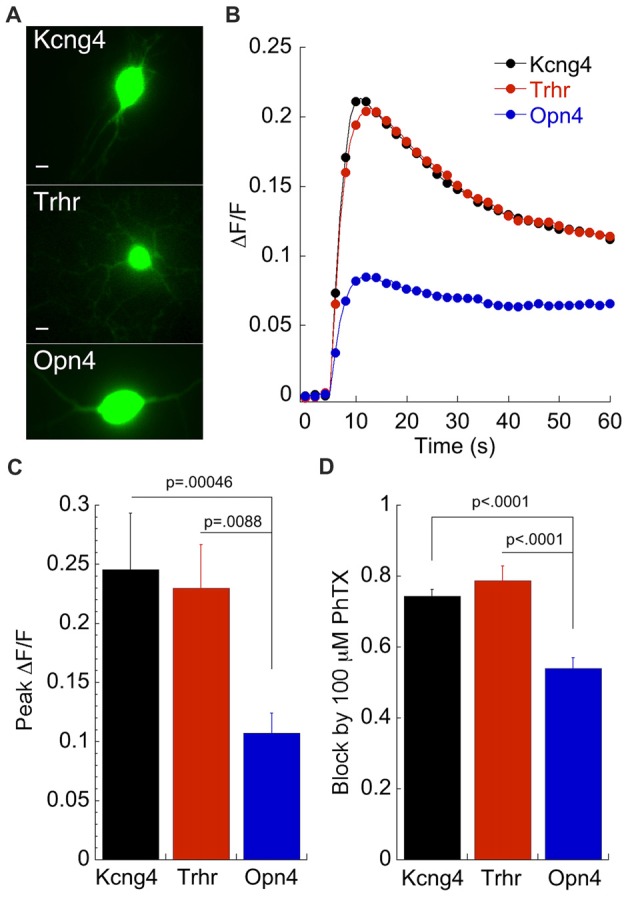
Calcium imaging of labeled RGCs reveals the heterogeneity of CP-AMPAR expression predicted by patch clamp recordings. **(A)** GFP expressing RGCs from each of the three mouse lines as indicated. Scale: 2.5 μm. **(B)** Averaged ΔF/F in response to 500 ms puffs of AMPA delivered at *t* = 5 s. Opn4: *n* = 30; Kcng4: *n* = 21; Trhr: *n* = 36. 100 μM Cd^2+^ is included to block voltage gated Ca^2+^ channels. **(C)** Average peak ΔF/F for the three cell types. **(D)** For comparison, block by 100 μM PhTX obtained using patch clamp recording from the same DIVs is also shown. Kcng4: *n* = 23; Trhr: *n* = 19; Opn4: *n* = 38.

Pharmacological data presented thus far predict a significantly higher Ca^2+^ influx through AMPARs in DS-RGCs and OFF alpha RGCs compared to ON alpha RGCs. To test this prediction, we measured Ca^2+^ transients associated with AMPA puffs in all three subtypes (Figure 6A). Both the mean time course of the transient for each RGC subtype (Figure 6B) and the peak ΔF/F (Figure 6C) verify this prediction. The peak ΔF/F of ON alpha RGCs was less than half of the ΔF/F measured in DSGC and OFF alpha RGCs, in good agreement with the predicted value based on the amplitude of the currents blocked by PhTX as described above (Figure 6D). Thus, both pharmacological and imaging data indicate a much lower Ca^2+^ permeability for AMPARs in ON α RGCs compared to these other subtypes. However, it is unclear if this distinction holds for RGCs in the intact retina at comparable stages of development.

**Figure 7:**
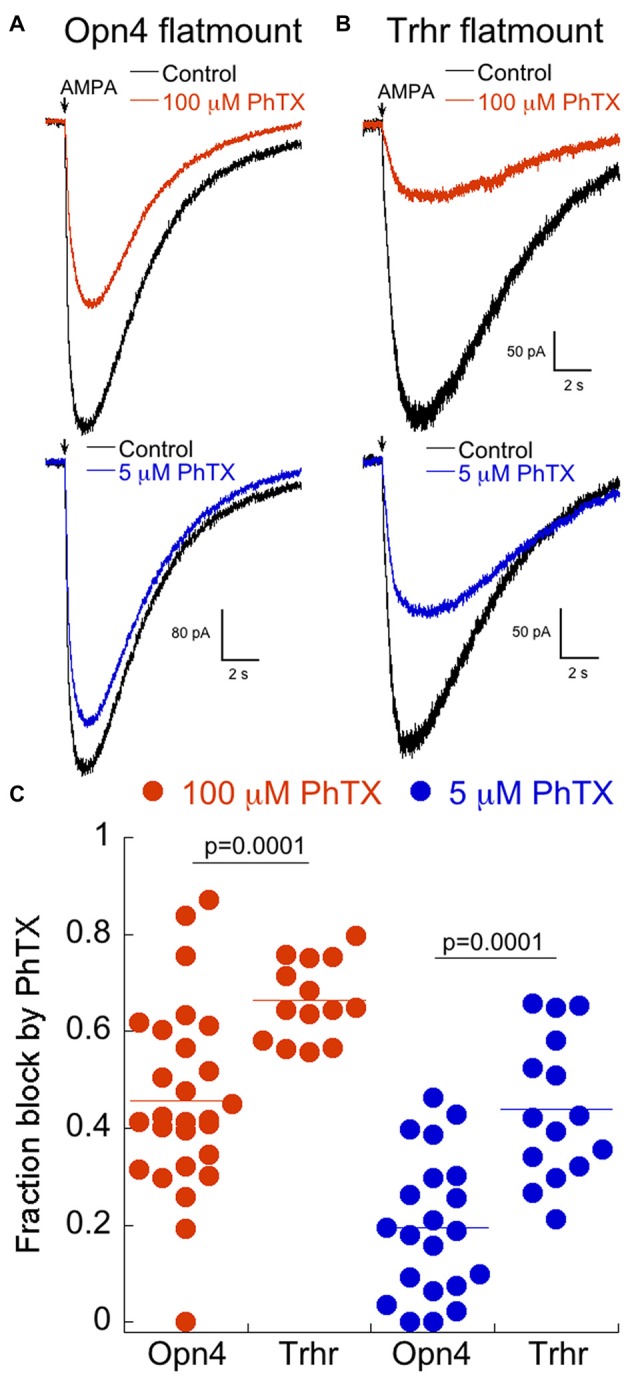
Labeled RGCs in Opn4 and Trhr retinas show a similar expression of CP-AMPARs as their counterparts in culture. **(A)** Example of the response to a puff of AMPA in the isolated retina of a labeled RGC from the Opn4 mouse line in 100 μM (top) or 5 μM PhTX (bottom). **(B)** Recording as in **(A)** from a labeled RGC from the Trhr mouse line. **(C)** Dot plot summary of the fraction of block by 100 and 5 μM PhTX in isolated retinas of the Trhr and Opn4 lines. Measurements were made from 18 to 23 day old mice.

Accordingly, we isolated retinas from Opn4 and Trhr mice between ages 19–26 days, and measured responses to puffs of AMPA in the absence or presence of 100 μM or 5 μM PhTX as described for cultured cells. Cadmium (100 μM) was always present to block synaptic transmission. In the Opn4 mouse, multiple subtypes of melanopsin-expressing cells are labeled, but we targeted those with the largest somas, which have previously been shown to be ON alpha RGCs (Estevez et al., 2012; Schmidt et al., 2014). Representative examples of the effects of 100 μM and 5 μM PhTX on the AMPAR current are illustrated in Figure 7. The amount of block observed in isolated retina with both concentrations of PhTX was consistently higher in ON-OFF DS RGCs from Trhr mice compared to ON α RGCs (Figures 7A,B). Results from individual RGCs are summarized in Figure 7C. It should be noted that the distribution of block was broader for ON α than for Trhr RGCs, particularly at 100 μM PhTX. Although the reason for this is unclear, it may reflect the presence of distinct compartments of AMPAR which may be differentially activated by AMPA puffs depending upon the precise location of the puffing pipet. Nevertheless, these data indicate that both classes of RGCs retain the same basic expression levels of AMPARs in the intact retina as they do in culture.

### ON Alpha and OFF Alpha RGCs Respond Differently to Elevated Pressure

**Figure 8:**
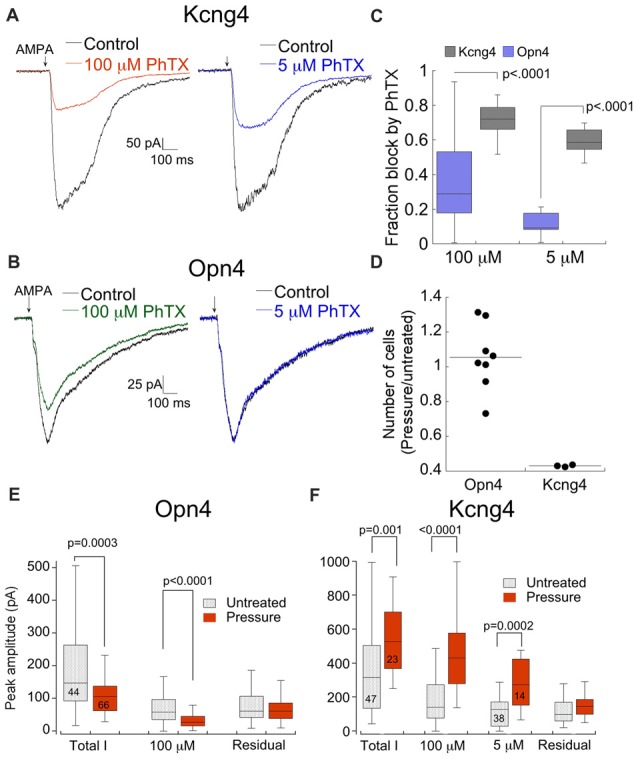
ON alpha and OFF alpha RGCs respond differently to the same stress. **(A)** Response of a fluorescent cell from the Kcng4 line to AMPA in the absence and presence of 100 μM and 5 μM PhTX after pressure treatment for 14 h. **(B)** As in **(A)**, except that the cell was from the Opn4 line. The culture was pressure-treated for 12 h. **(C)** Box and whisker plot summarizing the effects of elevated pressure on CP-AMPAR expression in RGCs from the Opn4 and Kcng4 lines. Opn4, 100 μM PhTX: *n* = 38; Opn4, 5 μM PhTX: *n* = 10; Kcng4, 100 μM PhTX: *n* = 14; Kcng4, 5 μM PhTX: *n* = 14. **(D)** Summary of RGC subtype specific survival 48 h following pressure treatment. Data are normalized by the ratio of the number of cells in pressure-treated and untreated cultures. Each dot represents one coverslip from pressure treated cells normalized to a randomly selected coverslip from sister untreated cultures. **(E**,**F)** Histogram comparing the effect of pressure on PhTX-sensitive and insensitive components of the AMPA current for Opn4 **(E)** and Kcng4 **(F)** RGCs. Number of sampled cells are indicated. Note that pressure selectively altered PhTX-sensitive, but not insensitive AMPA current.

Thus far we have established that RGCs express different ratios of CP- and CI-AMPARs both in culture and in the retina. Next we tested the possibility that RGCs respond differentially to elevated pressure. We examined the effects of pressure on OFF (Kcng4 line) and ON (Opn4 line) alpha RGCs, as ON-OFF DS-RGCs showed poor survival in culture even without elevated pressure. Both types of cells were subjected to 12 h of elevated pressure and CP-AMPAR expression was probed using PhTX as described above. Representative examples of the responses of both cell types to AMPA following pressure are shown in Figure 8. The response of OFF RGCs from the Kcng4 line was consistent with the overall RGC population. Block by both 100 μM and 5 μM PhTX was increased following 12 or more hours of elevated pressure, indicating a rise in the relative expression of CP-AMPARs (Figure 8A). Most notably, over 50% of the total current was blocked by the low concentration of PhTX (55 ± 3%), indicating that current flow was predominantly through homomeric CP-AMPARs (Figures 8A,C). ON alpha RGCs responded differently than OFF alpha RGCs and the overall RGC population. Following pressure treatment, 100 μM PhTX blocked AMPAR currents only modestly (Figures 8B,C), indicating that pressure decreased total CP-AMPAR expression. The effect on homomeric CP-AMPARs was even more dramatic, as 5 μM PhTX blocked little or none of the current. Thus, elevated pressure resulted in a reduction rather than an increase in CP-AMPAR expression in this cell type. Direct comparison of CP-AMPAR expression in both types of cells following pressure treatment shows a highly significant difference (Figure 8C). In addition to this difference in CP-AMPAR expression, there was a profound loss of OFF alpha RGCs 48 h after ceasing treatment, with only 43% of cells remaining compared to control, but there was no significant loss of ON alpha RGCs (103% of cells remained compared to control, Figure 8D). A change in the CP-AMPAR ratio following acute exposure to elevated pressure could result either from modulation of either CP- or CI-AMPARs. Measurements of the amplitude of both components clearly demonstrate a selective change in CP-AMPAR expression (Figures 8E,F). Pressure significantly reduced functional expression of CP-, but not CI-AMPAR in ON alpha RGCs, and increased CP-AMPAR expression in OFF RGCs. Importantly, pressure increased block by both 5 μM and 100 μM PhTX, indicating that labeled RGCs from the Kcng4 line increase both GluA2-lacking CP-AMPARs and heteromeric GluA2-containing receptor expression following exposure to acute pressure (Figure 8F). The differential response of these two RGCs populations underscores the specificity of the pressure stimulus.

## Discussion

Studies aimed at determining early events following elevation of IOP in animals have identified both morphological and functional changes in RGCs. Importantly, evidence from these studies indicates that OFF type RGCs are more vulnerable to both chronic and acute increases in IOP than ON RGCs (Della Santina et al., 2013; El-Danaf and Huberman, 2015; Ou et al., 2016; Della Santina and Ou, 2017). In particular, transiently responding OFF RGCs may be the most vulnerable. Here we investigated the possibility that RGC-specific changes in AMPA receptor expression contribute to this selective vulnerability, using an *in vitro* model system for acute elevation of IOP. Based on our findings, we conclude that: (1) RGC subtypes express distinct patterns of AMPA receptors that differ in their Ca^2+^ permeability. (2) In some RGC subtypes, but not others, elevation of ambient pressure triggers excessive increased expression of CP-AMPAR. (3) Amongst them are receptors classically considered to be Ca^2+^ impermeable, yet they contribute to Ca^2+^ influx; and (4) high levels of CP-AMPAR expression contribute to RGC death. Differences in Ca^2+^ influx through AMPARs has been observed previously in rat RGCs early in development (Rörig and Grantyn, 1993; Leinders-Zufall et al., 1994; Zhang et al., 1995).

### Identification of RGC Subtypes *in Vitro*

This study made use of the Opn4^Cre/+^ line as a source of ON alpha RGCs in culture. At least five different types of RGCs can be identified using this line (Ecker et al., 2010), including M4 ipRGCs that contribute to image forming vision and express nearly undetectable levels of melanopsin without amplification techniques (Estevez et al., 2012; Schmidt et al., 2014). Based on its size, response properties and immunoreactivity to SMI-32, an antibody against nonphosphorylated neurofilaments, the M4 ipRGC has been identified as the primary ON alpha RGC in the mouse retina (Schmidt et al., 2014). We found that the vast majority of fluorescent cells in the Opn4 line were highly immunoreactive for SMI-32, suggesting that ON alpha RGCs are the principal ipRGCs that survive under our culture conditions, but cannot rule out the possibility that other ipRGCs are present.

Unlike ON alpha RGCs, fluorescently labeled RGCs in culture from the Kcng4 line consistently responded to pressure by increasing CP-AMPARs. Although this line has been reported to label all alpha RGCs (Duan et al., 2014; Krieger et al., 2017), the number of labeled cells was much higher than the number of cells from the Opn4 line, indicating only a minor contribution of ON alpha RGCs. Furthermore labeled RGCs were only weakly reactive to SMI-32 (e.g., Figure 4C), and therefore may have been composed predominantly of OFF sustained alpha RGCs, which are not strongly labeled by this antibody (Ou et al., 2016). In addition, we failed to observe NMDA dependent plasticity in labeled Kcng4 cells, in agreement with a previous report (Jones et al., 2012). It is therefore tempting to speculate that cells labeled in the Kcng4 line are predominantly OFF type. Resistance to pressure-induced increases in CP-AMPAR expression on the part of ON alpha RGCs may contribute to their lack of vulnerability to elevated IOP demonstrated in previous studies. Conversely, high CP-AMPAR expression, particularly following transient elevation of pressure, may play a role in the susceptibility of OFF alpha RGCs demonstrated in these same studies.

### A Form of NMDA-Dependent Plasticity Is Conserved in Cultured ON RGCs

Our knowledge of glutamate receptor expression in ganglion cells is primarily confined to NMDA receptors, whose subunit composition in mature and developing retina, associated scaffolding proteins, and postsynaptic location vary according to RGC subtype (Chen and Diamond, 2002; Sagdullaev et al., 2006; Zhang and Diamond, 2009; Stafford et al., 2014). ON RGCs express NMDA receptors perisynaptically, requiring transmitter spillover for activation, while NMDARs of OFF RGCs are closer to release sites and appear to be stimulated by submaximal release (Sagdullaev et al., 2006; Zhang and Diamond, 2009). Consistent with this perisynaptic positioning, NMDA-dependent plasticity is observed in ON RGCs, but only following a regime of intense light stimulation, and is absent in OFF RGCs, studied in the intact retina (Jones et al., 2012). Interestingly in the present study we observe a similar NMDA-dependent plasticity in Opn4 cells, which are of the ON type, but not in Kcng4 cells. NMDAR activation drives insertion of CP-AMPARs as was reported previously, but only after 14 DIV, an age which correlates with synaptogenesis *in vivo* (Morgan et al., 2008). Thus some, if not all ON RGC subtypes maintain a pool of CP-AMPARs that can be rapidly delivered to the surface in response to changes in stimulus conditions, but are otherwise sequestered, perhaps to avoid excessive Ca^2+^ influx.

### How Many AMPAR Subtypes Are Modulated by Elevated Pressure?

The vast majority of AMPARs expressed in the brain are thought to exist as GluA2-containing heteromers, either GluA1/2 or GluA2/3 (Wenthold et al., 1996; Isaac et al., 2007; Lu et al., 2009), to be impermeable to Ca^2+^ (Hollmann et al., 1991; Burnashev et al., 1992b; Jonas and Burnashev, 1995), and display linear I–V relations (Verdoorn et al., 1991; Burnashev et al., 1992b) due to their insensitivity to block by intracellular polyamines (Bowie and Mayer, 1995; Donevan and Rogawski, 1995; Kamboj et al., 1995; Koh et al., 1995). Conversely, CP-AMPARs receptors are typically identified by an inwardly rectifying I–V relation due to intracellular block by polyamines, or extracellular block by low concentrations of polyamine toxins such as joro spider toxin, angiotoxin and PhTX (Burnashev et al., 1992a; Herlitze et al., 1993; Washburn and Dingledine, 1996; Bowie et al., 1999) rather than by direct measurements of Ca^2+^ permeability or influx. PhTX-74, an analog of PhTX used in the present study, differs from other toxins in that it blocks GluA2-containing AMPARs at higher concentrations (Nilsen and England, 2007; Poulsen et al., 2014). Imaging data presented in this study suggest that GluA2-containing AMPARs that are blocked by PhTX-74 exhibit significant permeability to Ca^2+^. The present study goes on to show that at least one population of RGCs, identified in the Kcng4^cre^ line, increases surface expression of both “classic” GluA2-lacking CP-AMPARs and GluA2-containing CP-AMPARs in response to elevated pressure. Without the use of PhTX-74, a change in the latter population would not have been detected.

What distinguishes GluA2-containing AMPARs that are blocked by PhTX-74 from those that are not? It is possible that AMPARs that are resistant to block might be composed of GluA2 homomers. However, AMPARs of this composition are thought to occur only rarely in the brain (Lu et al., 2009; Rossmann et al., 2011; Greger et al., 2017). Another possibility is that block is conferred by the presence of accessory proteins such as transmembrane AMPAR regulatory proteins (TARPs) and cornichons (Soto et al., 2007, 2009; Jackson and Nicoll, 2011). The presence of TARPs has been shown to increase block by PhTX-74 in both recombinant and native GluA2-containing AMPARs (Jackson et al., 2011).Yet another possibility is that elevated pressure changes the editing status of the Q/R site of newly inserted GluA2-containing AMPARs. Decreased levels of the RNA editing enzyme ADAR2 following elevated pressure *in vivo* has been reported previously (Wang et al., 2014). AMPARs with less than fully edited GluA2 subunits might be more susceptible to block by PhTX-74, providing a mechanism to account for the observation that the fraction of a PhTX-blocked GluA2-containing AMPARs increases after pressure treatment.

The presence of GluA2-containing, CP-AMPARs seem to be consistent with previous reports of AMPARs that are insensitive to polyamines such as spermine and the more widely used PhTX-433 and yet have significant permeability to Ca^2+^ (Gilbertson et al., 1991; Otis et al., 1995; Meucci et al., 1996; Meucci and Miller, 1998; Osswald et al., 2007), and seem to be particularly prevalent in the retina (Diamond, 2011; Bowie, 2012).

## Author Contributions

SN and WT: conceptualization. XW, WT and SN: methodology and writing-review and editing. XW, AC, CB and SN: investigation. SN: writing-original draft. WT and SN: supervision and funding acquisition.

## Conflict of Interest Statement

The authors declare that the research was conducted in the absence of any commercial or financial relationships that could be construed as a potential conflict of interest.
